# An investigation on removal of ciprofloxacin and norfloxacin by phycoremediation with an emphasis on acute toxicity and biochemical composition

**DOI:** 10.1038/s41598-023-41144-y

**Published:** 2023-08-25

**Authors:** R. Ricky, S. Shanthakumar

**Affiliations:** 1 Department of Environmental and Water Resources Engineering, School of Civil Engineering, Vellore Institute of Technology (VIT), Vellore, 632014 India; 2 Centre for Clean Environment, Vellore Institute of Technology (VIT), Vellore, 632014 India

**Keywords:** Pollution remediation, Environmental impact

## Abstract

Ciprofloxacin (CIP) and norfloxacin (NOR) belong to the class of emerging contaminants that are frequently detected in the aquatic environment as a binary mixture, responsible for the development of antibiotic-resistant genes and antibiotic-resistant bacteria. This study aims to investigate five different algal species *Chlorella vulgaris* (*Cv*), *Chlorella pyrenoidosa* (*Cp*), *Scenedesmus obliquus* (*So*), *Tetradesmus sp* (*T*) and *Monoraphidium sp* (*M*) for their tolerance and removal of binary mixture. The effects on biochemical composition in the algal species concerning the binary mixture and its removal efficiency are first reported in this study. The acute toxicity (96 h EC_50_) values are in the order of *So* > *Cp* > *T* > *M* > *Cv*, *Chlorella vulgaris* is the most sensitive algal species with 17.73 ± 0.24 mg/L and *Scenedesmus obliquus* is the least sensitive algal species with 39.19 ± 0.79 mg/L. The removal efficiency of the binary mixture was found to be in the order of *So* > *Cp* > *T* > *M* > *Cv*, *Scenedesmus obliquus* removed CIP (52.4%) and NOR (87.5%) with biodegradation as the major contributing removal mechanism. Furthermore, less toxic biotransformed products were detected in *Scenedesmus obliquus* and the biochemical characterization revealed that the growth-stimulating effect is higher with lipid (35%), carbohydrate (18%), and protein (33%) providing an advantage in the production of valuable biomass.

## Introduction

The worldwide consumption of antibiotics has increased from 635 to 674 units per 1000 population in developed countries and 1490 units to 1593 units in developing countries^1^. Studies have reported that 50–80% of the administered antibiotics are excreted through feces and urine as the parent compound and reach the aquatic environment, which has led to the development of antibiotic-resistant genes (ARGs) that have raised concerns in public health globally^2^. ARG has led to the death of 25,000 infants in European Union countries, 23,000 infants in the USA, and 58,000 infants in India every year^3,4^. Municipal wastewater is the major source of antibiotic pollution compared to hospital wastewater. The wastewater treatment plants (WWTPs) are not designed to eliminate these pollutants, as the metagenomic analysis has revealed that in WWTPs of 60 countries have a divergent distribution of ARGs^5–7^. India has the third largest active pharmaceutical industry fulfilling 50% of the generic medicine demand globally and is one of the top five pharmaceuticals consuming countries which is predicted to have an increase of 84% by 2030^8,9^. Fluoroquinolone (FQs) belongs to the third largest antibiotics accounting for 17% of the global market and it is an important class of antibiotics that has been extensively used to treat respiratory and urinary infections^10,11^. Ciprofloxacin (CIP) and norfloxacin (NOR) are the most frequently detected FQs in surface waters exceeding a 50% detection rate globally due to their excessive usage and it has been detected at different concentrations ranging from ng/L to µg/L^12^.

Various conventional and advanced treatment methods can be employed for the treatment of these pollutants but high operations and maintenance costs restrict their utilization in the municipal WWTPs. The incomplete mineralization of these contaminants generates transformed products that are toxic to the aquatic community and it has become a major concern^6^. Biodegradation by microorganisms is a good approach in the treatment of these pollutants with the advantage of environmental-friendliness and cost-effectiveness^12,13^. FQs are not easily biodegradable in conventional WWTPs but when treated with specific microbial species FQs can be biologically treated^14^. Algae are non-target organisms for antibiotics and are known to treat wastewater effectively removing nutrients, organic pollutants, and heavy metals with the advantage of biomass production^6,15^. CIP and NOR are tested for phycoremediation and studies have shown the ability of algal species to biodegrade the pollutants individually^16–19^. *Chlorella vulgaris* (*Cv*), *Chlorella pyrenoidosa* (*Cp*), and *Scenedesmus obliquus* (*So*) have shown their potential in removing CIP, 85% (*Cv*), 79% (*Cp*), and 75% (*So*) and for NOR, 52% (*Cv*), 50% (*Cp*), and 43% (*So*) with initial concentration CIP (0.029 µg/L) and NOR (0.032 µg/L) separately^20^. CIP and NOR have been detected in the Indian surface waters as a binary mixture with the maximum detected concentration of 6.5 mg/L (CIP) and 0.52 mg/L (NOR)^21^.

Individual toxicity assessment studies have reported that an increase in the concentration of pollutants will inhibit the growth of algae and this will have an impact on their removal and biochemical composition^16,22^. EC_50_ (concentration at which 50% of the growth rate of algae is inhibited) is the most accepted evaluation for toxicity assessment in algal species^23^. There are also other algal species such as *Tetradesmus sp* (*T*) and *Monoraphidium sp* (*M*) that requires much attention in phycoremediation studies for the removal of antibiotics. *Tetradesmus sp* (*T*) and *Monoraphidium sp* (*M*) have shown good phycoremediation properties and have the potential to produce valuable biomass with good biochemical properties^24–27^, but both the species have yet not been tested for their potential in the removal of FQs. Phycoremediation potential can be stunned when the test values are greater than EC_50_. Therefore, a growth inhibition test must be performed before employing the algae for the removal of pollutants, as toxic pollutants are known to inhibit the growth of algae. These results will contribute to the FQs toxicity data on these selected species and the application of these species in wastewater treatment plants can be identified.

Most of the studies have focused on the individual EC_50_ assessment and removal of pollutants, without any information regarding its effects on biochemical composition (proteins, carbohydrates, and lipids)^16,17,28,29^. In the case of a binary mixture (CIP+NOR) acute toxicity assessment, removal, and its biotransformed products have not yet been reported, as the combination of these pollutants exists in the environment. With this in view, this study aims to screen and select the most effective and tolerant algal species among the five different algae representing four different genera (*Cv, Cp, So, T,* and *M*), to assess the acute toxicity of binary mixtures and its effects on biochemical composition (proteins, carbohydrates, and lipids) in the selected algal species, and to investigate the removal potential of the binary mixture (CIP+NOR) and the mechanisms adapted by the algal species for their removal, biotransformed products, and its effects on biochemical composition (proteins, carbohydrates, and lipids).

## Materials and methods

### Fluoroquinolones

Stock solutions of norfloxacin (NOR) (CAS No: 70458-96-7) and ciprofloxacin (CIP) (CAS No: 85721-33-1) with > 98% purity (HPLC grade) were prepared by dissolving CIP and NOR in the ultrapure water and stored in an airtight Schott glass container at 4 °C in dark conditions for no longer than 7 days.

### Determination of fluoroquinolones concentration

CIP and NOR concentrations were determined according to the USP 28-NF 23 s supplement using 844 UV–VIS compact ion chromatography equipped with an HPLC column (Hichrom-Alltima 5 µm C-18 with dimensions 250 × 4.6 mm)^30–32^. Acetonitrile (15%) and ultrapure water (85%) with pH = 3.2 were used as the mobile phase with a flow rate adjusted to 1 mL/min and 250 µL injection volume^19^. The wavelength of the UV detector was set for 280 ± 5 nm which corresponds to the maximum wavelength for both the antibiotics and retention time for NOR was 14.5 min and for CIP 16.5 min, respectively. The limit of detection for CIP and NOR in this study was 0.03 µg/L and 0.02 µg/L, respectively. The concentration was estimated using IC-NET 2.3 software integrated with HPLC equipment and upon the completion of the analysis, the consolidated overlay curves of the individual analysis were plotted using the IC-NET software. The biotransformed products were identified using Liquid chromatography-electron spray ionization tandem mass spectrometry. Formic acid (0.1%) and acetonitrile at a volumetric ratio (95:5) is used as mobile phase. Mass spectrometer conditions and the column details are presented elsewhere^33^.

### Algal species and culture conditions

*Chlorella vulgaris* (*Cv*) (BDU-GD003) was procured from the National Repository for Microalgae and Cyanobacteria (NRMC), Bharathidasan University, Tamilnadu, India. *Chlorella pyrenoidosa* (*Cp*) (NCIM-2738)*, Scenedesmus obliquus* (*So*) (NCIM-5586), *Tetradesmus sp* (*T*) (NCIM-5797), and *Monoraphidium sp* (*M*) (NCIM-5792), were procured from the National Collection of Industrial Microorganisms (NCIM), Pune, India. All the procured cultures were sub-cultured in BG-11 medium under sterile conditions in an algal growth chamber with the light intensity of 50 µmol photon/m^2^/s in a 12 h light and dark cycle at 27 °C until their growth phase was attained.

### Determination of algal growth

Cell density (cells/mL) was measured using Neubauer’s improved hemocytometer under 40 × trinocular microscope magnification and the specific growth rate (µ) was calculated using the following Eq. (1)^34^.

1 $$ \upmu { }\left( {d^{ - 1} } \right) = \ln \left( {C_{f} } \right) - \ln \left( {C_{i} } \right)/t_{f} - t_{i} $$

where C_i_ and C_f_ are the cell density measured at the initial time (t_i_) and final time (t_f_).

### Extraction and analysis of chlorophyll-a, chlorophyll-b, and carotenoid

2 mL of algal cells were harvested by centrifuging the cells at 5000 rpm for 10 min from the experimental runs. The supernatant was discarded and resuspended with 5 mL of 90% methanol and incubated at 60 °C for 5 min and then centrifuged again for 10 min. The supernatants were collected in separate tubes and optical densities were measured for 665, 652, and 470 nm wavelength using a UV–visible spectrophotometer, and the concentration of the extracted pigment was calculated using the following Eqs. (2)–(4)^16^.

2 $$ Chlorophyll{ - }a \left( {chl{ - }a} \right)\left( \frac{mg}{L} \right) = 16.82\, A_{665} - 9.28 \,A_{652} $$

3 $$ Chlorophyll{ - }b \left( {chl{ - }b} \right)\left( \frac{mg}{L} \right) = 36.92 \,A_{652} - 16.54\, A_{665} $$

4 $$ Carotenoid\left( \frac{mg}{L} \right) = \frac{{1000 \,A_{470} - 1.91\, \left( {chl{ - }a} \right) - 95.15\, \left( {chl{ - }b} \right)}}{225} $$

### Determination of biochemical composition

Protein, carbohydrates, and lipids are the three important biochemical compositions of algae. Samples collected from the experimental runs were subjected to centrifugation to extract the cell pellets from the algal medium^35^. The harvested cell pellets were subjected to the lowry method to extract the protein from the harvested cell pellets and quantified using bovine serum albumin as standards in a UV–visible spectrophotometer^36^.The Anthrone method was used to extract the carbohydrates from the harvested cell pellets and quantified using glucose as standards in a UV–visible spectrophotometer^36^. Lipids were extracted using the sonication method and quantified gravimetrically^36,37^. Dry cell weight (DCW) was calculated gravimetrically by drying the harvested cell pellets. Lipids, protein, and carbohydrates were expressed on a percentage basis from the harvested biomass using the following Eqs. (5)–(7)^35^.

5 $$Lipids\, \left(\%\right)=\frac{Lipids\left(\frac{mg}{L}\right)}{DCW \left(\frac{mg}{L}\right)} \times 100$$

6 $$Protein\, \left(\%\right)=\frac{Protein\left(\frac{mg}{L}\right)}{DCW \left(\frac{mg}{L}\right)} \times 100$$

7 $$Carbohydrates\, \left(\%\right)=\frac{carbohydrates\left(\frac{mg}{L}\right)}{DCW \left(\frac{mg}{L}\right)} \times 100$$

### Growth inhibition test

Acute toxicity experiments were conducted according to OECD 201 guidelines^23^. Different concentrations of CIP and NOR ranging between 1 and 100 mg/L were used for individual tests, and CIP+NOR equal proportions of fixed concentrations ratio of (1:1) ranging between 1 and 50 mg/L were spiked into the 100 mL algal medium. The initial cell density for all the experiments was maintained at 100 × 10^4^ cells/mL. Three replicates of test runs (with pollutants) and control runs (without pollutants) were incubated in the algal growth chamber equipped with an orbital shaker (120 rpm) for the growth inhibition test. Percentage inhibition (%I) was calculated using the following Eq. (8)^38^.

8 $$\%\, \mathrm{I}=\frac{\left(\mu c- \mu t\right)}{\mu c} \times 100$$

where $\mu c$ and $\mu t$ are the cell density measured in the control run and test run.

EC_50_ is the effective concentration that inhibits 50% of the algal growth. A dose–response curve was plotted using percentage inhibition (%I) calculated for different concentrations, to find EC_50_ linearising the response data is needed to perform the regression analysis, Probit statistical analysis was performed to linearise the data as per OECD 201 guidelines^23^ to calculate EC_50_. The EC_50_ values of biotransformed products were predicted using ECOSAR software (version 2.2)^39^.

### Risk quotients assessment of binary mixture

Risk quotients (RQs) try to estimate the potential risk for algae caused by the environmental detected maximum concentration. RQ is calculated by the following Eqs. (9) and (10) ^40^.

9 $$\mathrm{RQ }=\frac{\mathrm{MEC}}{PNEC}$$

10 $$PNEC=\frac{{EC}_{50}}{1000}$$

where MEC is measured environmental concentration, PNEC is predicted no effect concentration.

When calculated

RQ > 1 indicates that there is an ecotoxicological risk for the algal species.

RQ < 1 indicates there is no risk for the algal species.

### Determination of fluoroquinolones removal mechanism in algal cells

A series of experimental runs were performed with five different algal species with control runs (without CIP + NOR) and test runs (with CIP + NOR) in 250 mL Erlenmeyer flasks containing 100 mL of algal medium with an average initial cell density of 100 × 10^4^ cells/mL. CIP (6.5 mg/L) and NOR (0.5 mg/L) were spiked in the test runs. Duplicate experimental runs were incubated in the algal growth chamber equipped with an orbital shaker (120 rpm) with the light intensity of 50 µmol photon/m^2^/s in a 12-h light and 12-h dark cycle at T = 30 °C ± 1 °C until the growth phase declined to death phase. 5 mL of sample is collected from the experimental runs and centrifuged for 10 min at 2500 rpm for the determination of CIP+NOR total removal is calculated by the following Eq. (11)^19^.

11 $$Total\, removal \left(\%\right)={(C}_{i}-{C}_{f})/{C}_{i}\times 100$$

where C_i_ is the initial concentration of (CIP+NOR) in the medium and C_f_ is the residual concentration of (CIP+NOR).

Bioadsorption, bioaccumulation, and biodegradation are the three major removal mechanisms adopted by algae in the removal of antibiotics^41^. Bioadsorption (R_ad_) is determined using the high-speed centrifuging method by increasing the rpm from 2500 to 5000 rpm without disrupting the cell wall^19,42,43^. The collected supernatant was used for the determination of R_ad_. Bioaccumulation (R_ac_) is determined using the ultrasonication method^19,44^. After 30 min of sonication, the sample is centrifuged for 10 min at 2500 rpm and the supernatant was used for the determination of R_ac_. Biodegradation (R_b_) is calculated using the following Eq. (12)^19^.

12 $${R}_{b}\left(\%\right)={(C}_{i}-{C}_{f}- {R}_{ad}-{R}_{ac}-abiotic\, removal)/{C}_{i}\times 100$$

Abiotic removal (photodegradation) is determined by keeping the control runs without algae in light and dark conditions for a period of 7 days) and is calculated using the following Eq. (12). Since FQs are known to be photosensitive compounds Eq. (13)^17,19,45^.

13 $$Abiotic\, removal \left(\%\right)=\left(\frac{\left(CIP+NOR\right)dark-\left(CIP+NOR\right)light}{{C}_{i}}\right)\times 100$$

**Figure 1:**
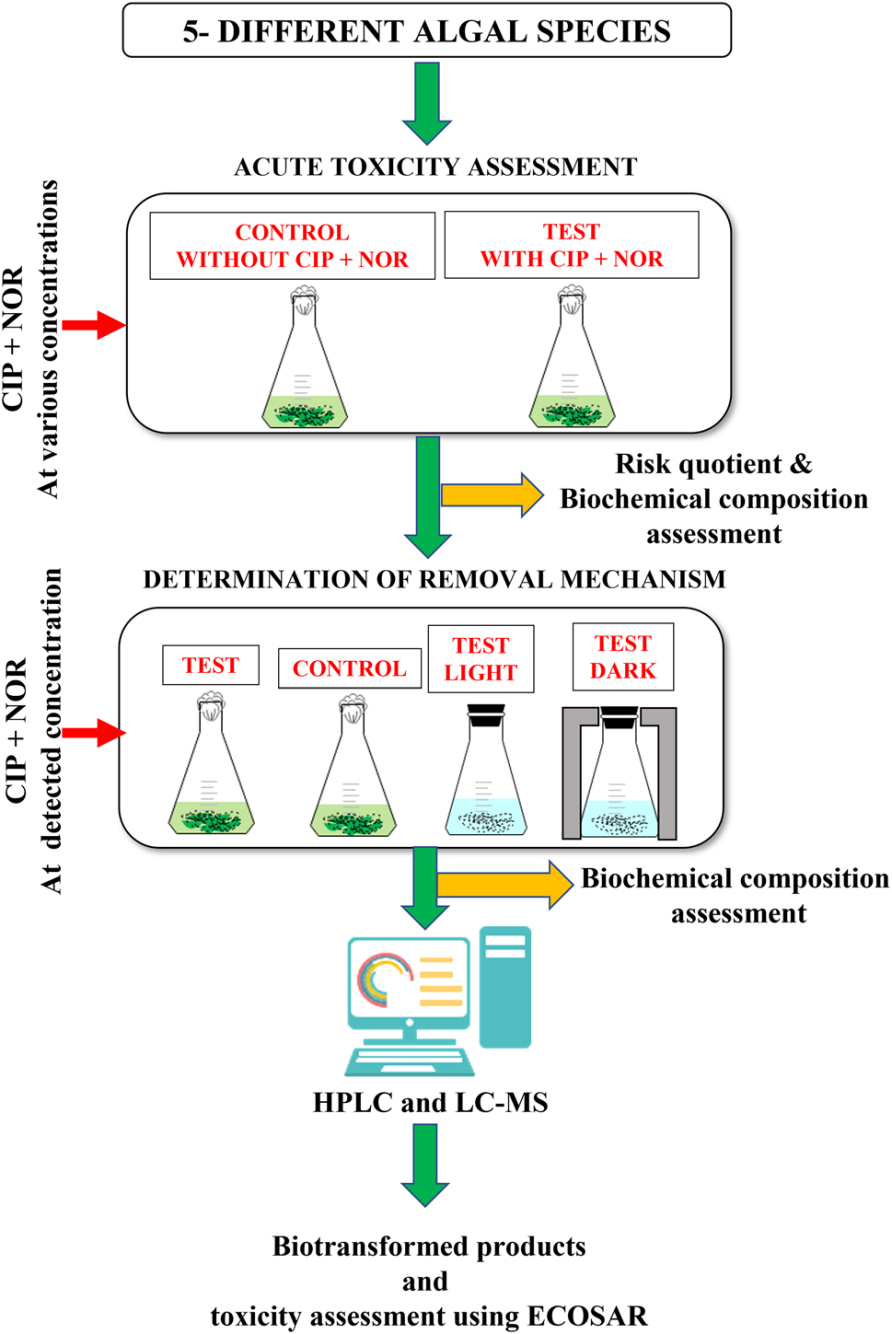
Methodology followed in this study.

Figure 1 depicts the overall methodology followed in this study.

### Statistical analysis and plotting

The growth inhibition study was conducted in triplicates and the Probit analysis for EC_50_ was calculated using SPSS software (version 26). The removal study was conducted in duplicates and the mean values were presented as results. One-way ANOVA coupled with the Dunnett test was conducted to find the statistical significance (*p* < 0.05) between the control runs and the test runs in the experimental study. The statistical analysis and graph plotting were carried out using origin pro software (version 9.9). The chemical structures were depicted using Chemdraw (version 20.0).

## Results and discussion

### Acute toxicity and risk quotients assessment of CIP and NOR

**Figure 2:**
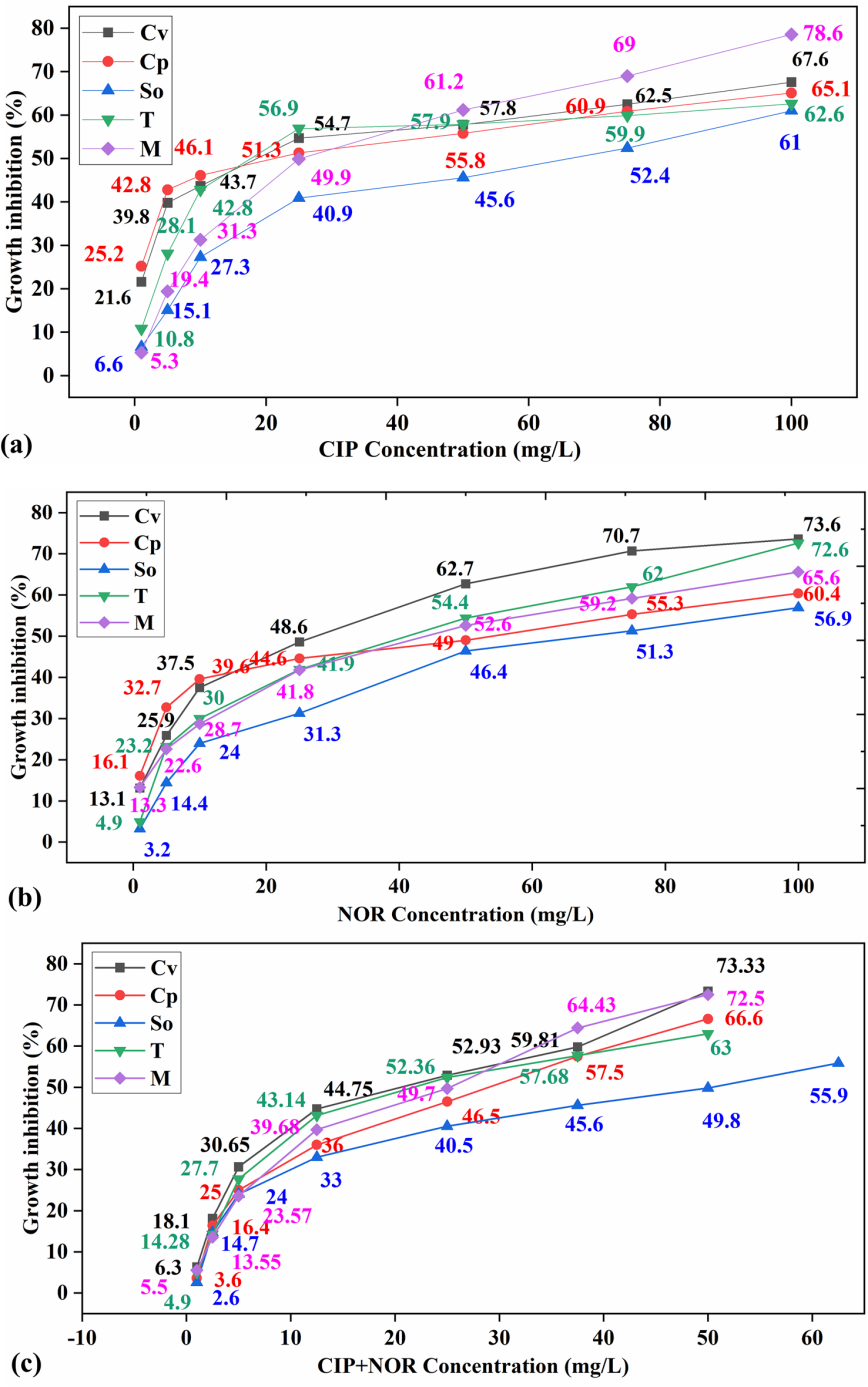
Dose—Growth inhibition (%) response curve for (**a**) CIP, (**b**) NOR, (**c**) CIP+NOR for algal species.

Acute toxicity assessment of CIP, NOR, and binary mixture (CIP+NOR), upon the selected algal species was conducted to understand the growth stimulatory and inhibitory effects that a target pollutant can cause individually and in combined form. Figure 2 depicts the dose–response curve for CIP, NOR, and CIP+NOR. The highest growth inhibition for CIP was recorded for *Monoraphidium sp (M)* 78.6% followed by 67.6% (*Cv*), 65.1% (*Cp*), 62.6% (*T*), and 61% (*So*). For NOR, the highest growth inhibition of 73.6% was recorded for *Chlorella vulgaris *(*Cv*) followed by 72.6% (*T*), 65.6% (*M*), 60.4% (*Cp*), and 56.9% (*So*). For binary mixture, the highest growth inhibition was observed in *Chlorella vulgaris *(*Cv*) 73.33% followed by 72.5% (*M*), 66.6% (*Cp*), 63% (*T*), and 49.8% (*So*). EC_50_ values were calculated using the Probit statistical analysis from the experimental runs (CIP, NOR, and CIP + NOR) and tabulated in Table 1. The 96 h EC_50_ values are in the order of (*So*) > (*T*) > (*M*) > (*Cp*) > (*Cv*) for CIP, (*So*) > (*Cp*) > (*M*) > (*T*) > (*Cv*) for NOR, and (*So*) > (*Cp*) > (*T*) > (*M*) > (*Cv*) for CIP+NOR*.* The higher the EC_50_ greater their tolerance towards the specific pollutant. From the EC_50_ values, the PNEC and RQs were calculated and tabulated in Table 1.

**Table 1:** Risk quotient assessment of CIP, NOR, and CIP+NOR for (*Cv*)*,* (*Cp*)*,* (*So*)*,* (*T*), and (*M*) and EC_50_ values corresponding to mean ± SD (n = 3).

Algal species	Antibiotic	EC_50_ (mg/L)	EC_50_ categorization	PNEC (mg/L)	RQ	RQ categorization
*Cv*	CIP	18.29 ± 0.47	Harmful	0.01829	355.39	High risk
	NOR	21.97 ± 0.44	Harmful	0.02197	23.67	High risk
	CIP+NOR	17.73 ± 0.24	Harmful	0.017734	395.85	High risk
*Cp*	CIP	18.45 ± 0.5	Harmful	0.01845	352.30	High risk
	NOR	40 ± 1.7	Harmful	0.04	13.00	High risk
	CIP+NOR	24.26 ± 0.37	Harmful	0.024259	289.38	High risk
*So*	CIP	55.72 ± 1.2	Harmful	0.05572	116.65	High risk
	NOR	63.83 ± 0.42	Harmful	0.06383	8.15	High risk
	CIP+NOR	39.19 ± 0.79	Harmful	0.0391917	179.12	High risk
*T*	CIP	26.47 ± 0.8	Harmful	0.02647	245.56	High risk
	NOR	33.74 ± 0.86	Harmful	0.03374	15.41	High risk
	CIP+NOR	21.63 ± 1.26	Harmful	0.0216313	324.53	High risk
*M*	CIP	25.73 ± 0.65	Harmful	0.025728	252.64	High risk
	NOR	39.61 ± 0.66	Harmful	0.03961	13.13	High risk
	CIP+NOR	19.52 ± 0.23	Harmful	0.019523	359.58	High risk

The Commission of the European Communities (EU directive 93/67/EEC) classifies the pollutant in the “harmful” category to the aquatic organism when the calculated EC_50_ value falls within the range of (10–100 mg/L)^46^. While categorizing the algal species according to calculated EC_50_ values and RQ values, results indicate that CIP+NOR is harmful and possess a high risk to the algal species. The individual acute toxicity assessment results also affirm that it poses a threat to the algal species even when they are present in a separate form. Studies conducted for EC_50_ growth inhibition test for CIP and NOR also affirm that the values fall under the harmful category for *Chlorella vulgaris* (CIP-96 h EC_50_ < 100 mg/L)^47,48^ and (NOR-72 h EC_50_ < 100 mg/L)^49^.

The inhibitory effects increased acute toxicity assessment of CIP+NOR increased due to the combined toxicity. A study performed by Magdaleno et al.^40^ reported that growth inhibition caused by the binary mixture (ciprofloxacin and cephalothin) for the algae *Pseudokirchneriella subcapitata* is greater than the individual growth inhibition and the results obtained in this study is in line with the reported literature.

The response to the pollutants in algal species is known to vary according to their physiology, morphology, cytology, and phylogenetics characteristics^22^. The inhibitory effects varied according to the species and their tolerance toward the pollutants, as the binary mixture had a serious impact on their inhibition levels. *Scenedesmus obliquus (So)* is the least sensitive algal species to the toxic effects of antibiotics (CIP, NOR, and CIP+NOR) whereas, *Chlorella vulgaris (Cv)* is the most sensitive species among the selected algal species. No toxicity data for *Chlorella pyrenoidosa (Cp), Scenedesmus obliquus (So), Tetradesmus sp (T),* and *Monoraphidium sp (M)* have been reported for CIP, NOR, and CIP+NOR. Therefore, these results contribute to the FQs toxicity data on these selected species.

### Effect of CIP+NOR on biochemical composition in algal species

#### Effect on pigments (chl-a, chl-b, and carotenoids)

**Figure 3:**
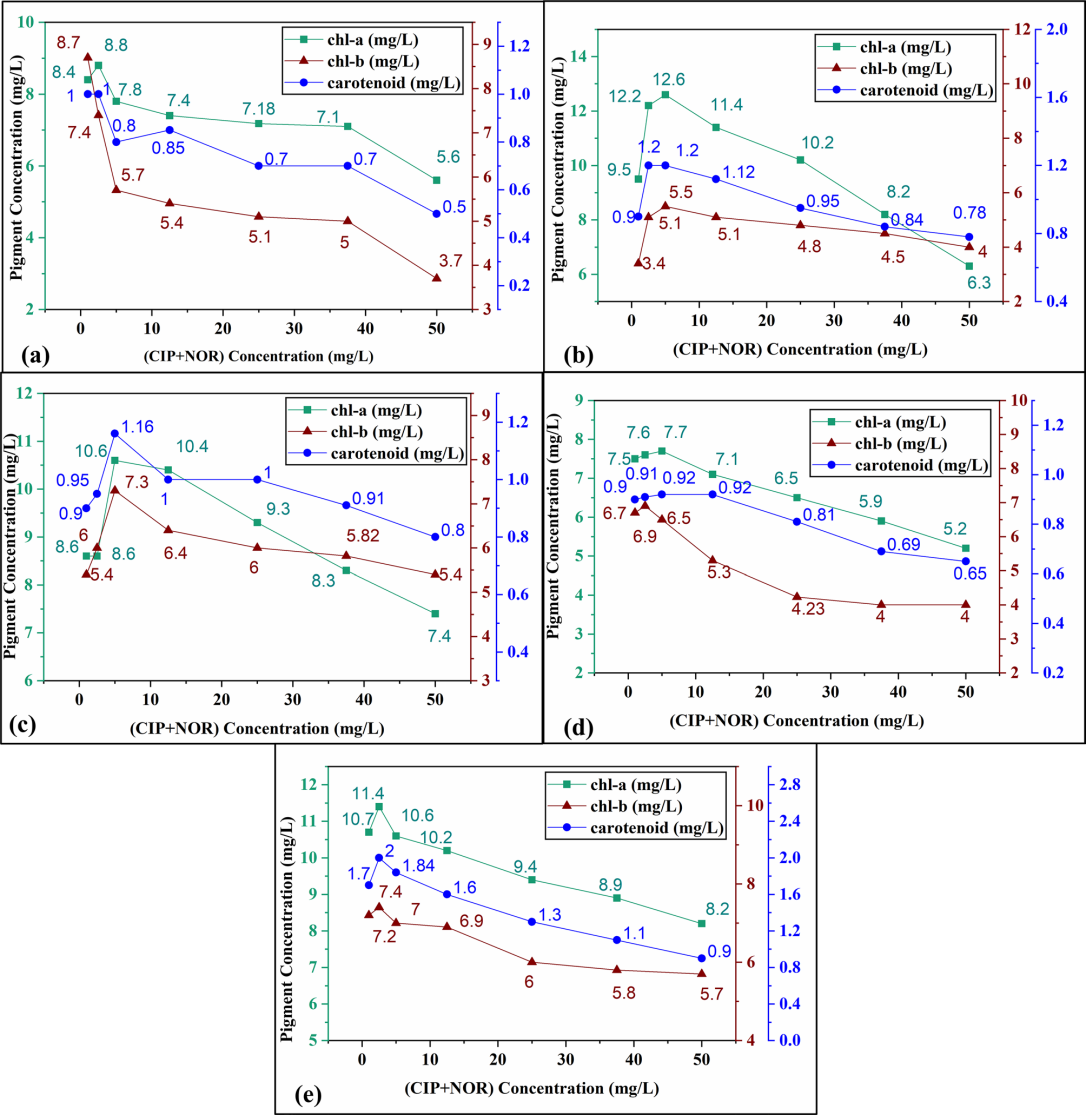
Effect of the binary mixture (CIP+NOR) on chl-a, chl-b, and carotenoids for (**a**) *Cv*, (**b**) *Cp,* (**c**) *So,* (**d**) *T,* and (**e**) *M.*

Chlorophyll is a major light-harvesting pigment involved in photosynthesis and exposure to pollutants is known to alter the chl-a and chl-b synthesis^47,50^. During the exposure of algae to environmental stress, carotenoids are known to serve as a photosystem protective mechanism and it plays a crucial role in quenching the singlet oxygen by deactivating the active chlorophyll^51^. The extracted chl-a, chl-b, and carotenoid pigments from the algal species were plotted to study the binary effects of CIP+NOR upon pigments at different concentrations as shown in Fig. 3. The effect varied according to the sensitivity of the algal species as well as the concentration of the pollutants. It is observed that in the algal species *Chlorella pyrenoidosa *(*Cp*) and *Scenedesmus obliquus (So),* there is an increase in pigment activity at concentrations < 10 mg/L (Fig. 3b,c) and the carotenoids concentration increased up to their corresponding EC_50_ levels as a response to the stress and then significantly decreased to 11% (*So*) and 13% (*Cp*) at 50 mg/L concentration indicating the damage to the algal cells as the concentration increases.

In *Tetradesmus sp* (*T*) the same activity in pigments was observed up to 5 mg/L (Fig. 3d) and the carotenoid contents decreased up to 27% at 50 mg/L concentration. In *Monoraphidium sp* (*M*) increased pigment activity was observed at concentrations < 2.5 mg/L (Fig. 3e) and the carotenoid contents decreased up to 47% at 50 mg/L concentration and for *Chlorella vulgaris* (*Cv*), increased chl-a and chl-b activity was observed at 1 mg/L (Fig. 3a), being the most sensitive algal species among the selected algal species. The carotenoid concentration gradually decreased up to 50% at 50 mg/L concentration indicating damage to the cells. The results of the present study are consistent with the studies carried out on *Chlamydomonas mexicana*^16^, *Chlamydomonas reinhardtii*^51^*,* and *Scenedesmus obliquus*^42^ which affirms that toxic stress can alter pigment activity according to their exposed concentration in the medium and hinder the photosynthetic activity in algal cells.

#### Effect on protein, carbohydrates, and lipids

**Figure 4:**
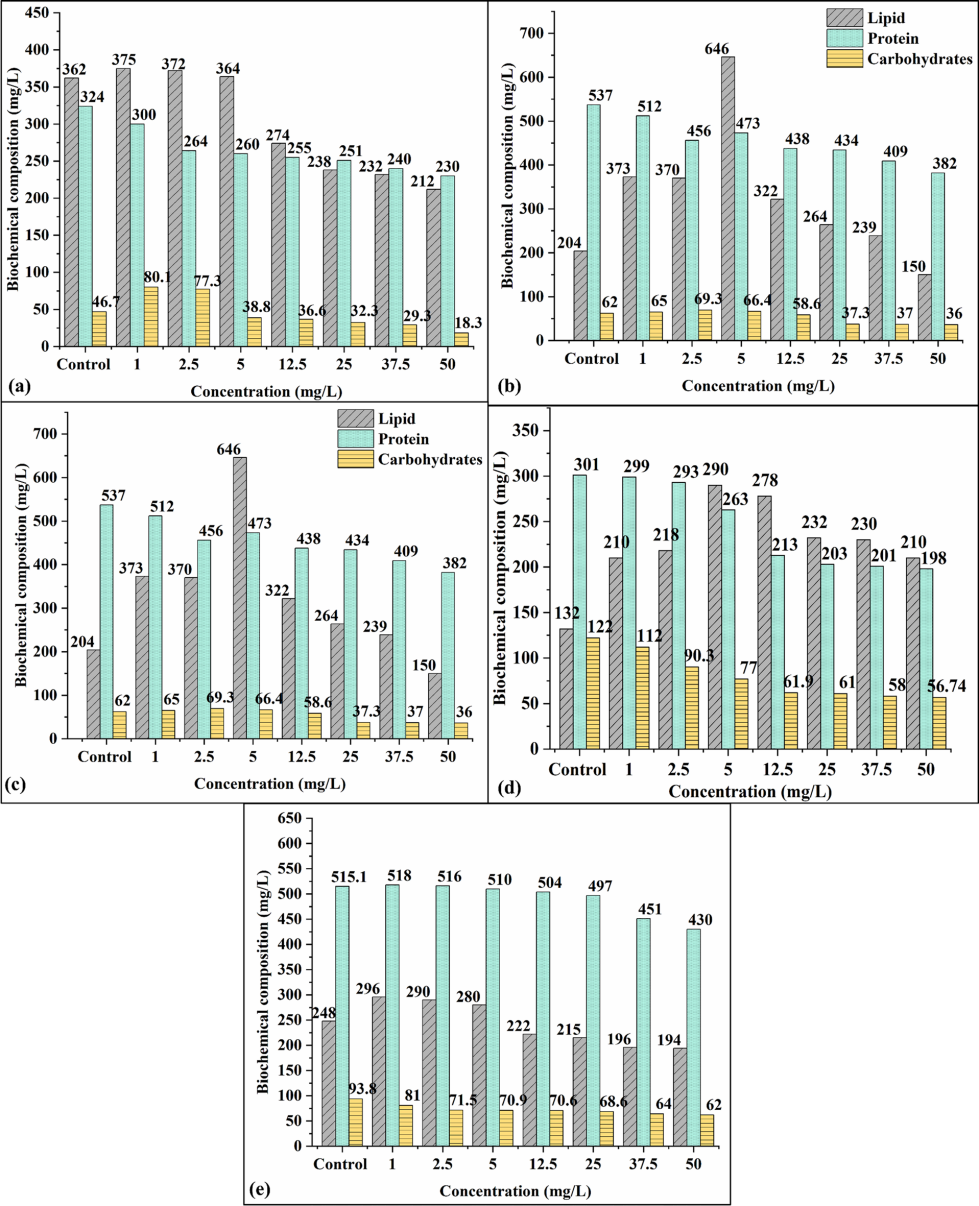
Effect of the binary mixture (CIP+NOR) on protein, lipids, and carbohydrates for (**a**) *Cv*, (**b**) *Cp,* (**c**) *So,* (**d**) *T,* and (**e**) *M.*

After 96 h exposure to the various concentration of CIP+NOR to the selected algal species, the secretion of the protein, lipid, and carbohydrate concentration varied according to the algal species. Figure 4 depicts the effect of different concentrations of the binary mixture (CIP+NOR) on the protein, lipid, and carbohydrates in the selected algal species (*Cv*, *Cp*, *So, T,* and *M*) to their control runs respectively. The growth-stimulating effects were observed in all the selected algal species within their calculated 96 h EC_50_ value. For *Chlorella vulgaris* (*Cv*) and *Chlorella pyrenoidosa (Cp),* growth-stimulating effects were observed below their EC_50_ value (< 25 mg/L). Inhibitory effects were observed when concentration increased beyond the EC_50_ value. The accumulation of protein has decreased drastically in all the concentrations compared to the control runs. Maximum lipid accumulation for *Chlorella vulgaris* (375 mg/L) was reported at 1 mg/L and for *Chlorella pyrenoidosa* (646 mg/L) at 5 mg/L. In our previous study^19^, when *Chlorella vulgaris* was exposed to CIP (5 mg/L), growth-stimulating effects were observed with higher accumulation of lipids (465 mg/L), carbohydrates (39 mg/L), and protein (608 mg/L) than reported in this study. The decrease in the biochemical composition in this study is attributed to the binary mixture toxicity.

When *Scenedesmus obliquus (So) was* exposed to the binary mixture*,* the lipid concentration increased up to 319 mg/L and then decreased beyond the EC_50_ concentration (39 mg/L). An increase in protein concentration was observed at 1 mg/L and 2.5 mg/L and then reduced drastically compared to control runs, whereas for carbohydrates the value decreased in all test runs. For *Tetradesmus sp* (*T*), there is a gradual decrease in protein and carbohydrates when the CIP+NOR concentration increases but lipid accumulation was higher in all the concentrations. The maximum lipid accumulation of 290 mg/L was observed at 5 mg/L (CIP+NOR). In the *Monoraphidium sp* (*M*) species, at 1 mg/L of CIP+NOR concentration, maximum accumulation of lipids (296 mg/L) and proteins (518 mg/L) was observed, whereas for carbohydrates the levels dropped in all the test runs. The reason for this behaviour in algal cells is due to the fact that during photosynthesis chlorophyll pigment performs carbon fixation and stores energy in the form of starch, but when the photosynthetic activity of the algae decreases the carbohydrate production also decreases. The observed results in this present study are consistent with the previous report on antibiotic stress in algae^52^.

### Removal of the binary mixture (CIP+NOR)

**Figure 5:**
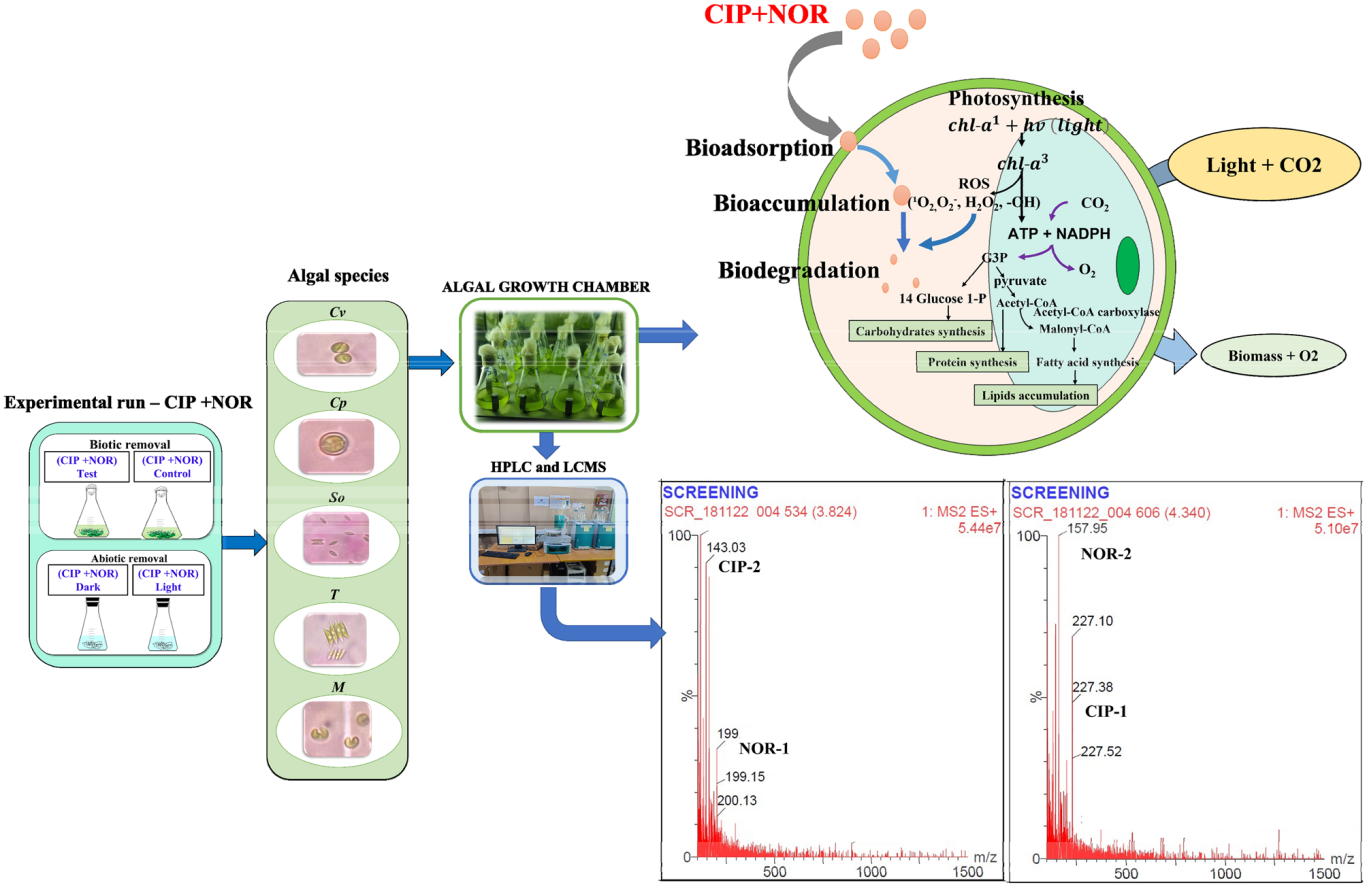
Experimentation for the removal of CIP+NOR.

A binary mixture of CIP and NOR with concentrations of 6.5 mg/L (CIP) and 0.5 mg/L (NOR) is selected for the removal studies as this binary mixture at these concentrations is detected in the Indian aquatic environment^21^. The EC_50_ value is known to increase when the exposure time is increased as algae are known to acclimatize the toxic conditions to survive in the aquatic environment^16^. From the acute toxicity results, we can conclude that all the selected algal species can tolerate this selected concentration of the binary mixture, growth-stimulating effect in algae can enable the algal species to remove the binary mixture from the algal medium. Figure 5 depicts the experimentation followed for the removal of CIP+NOR.

#### Effect of CIP+NOR on algal growth

**Figure 6:**
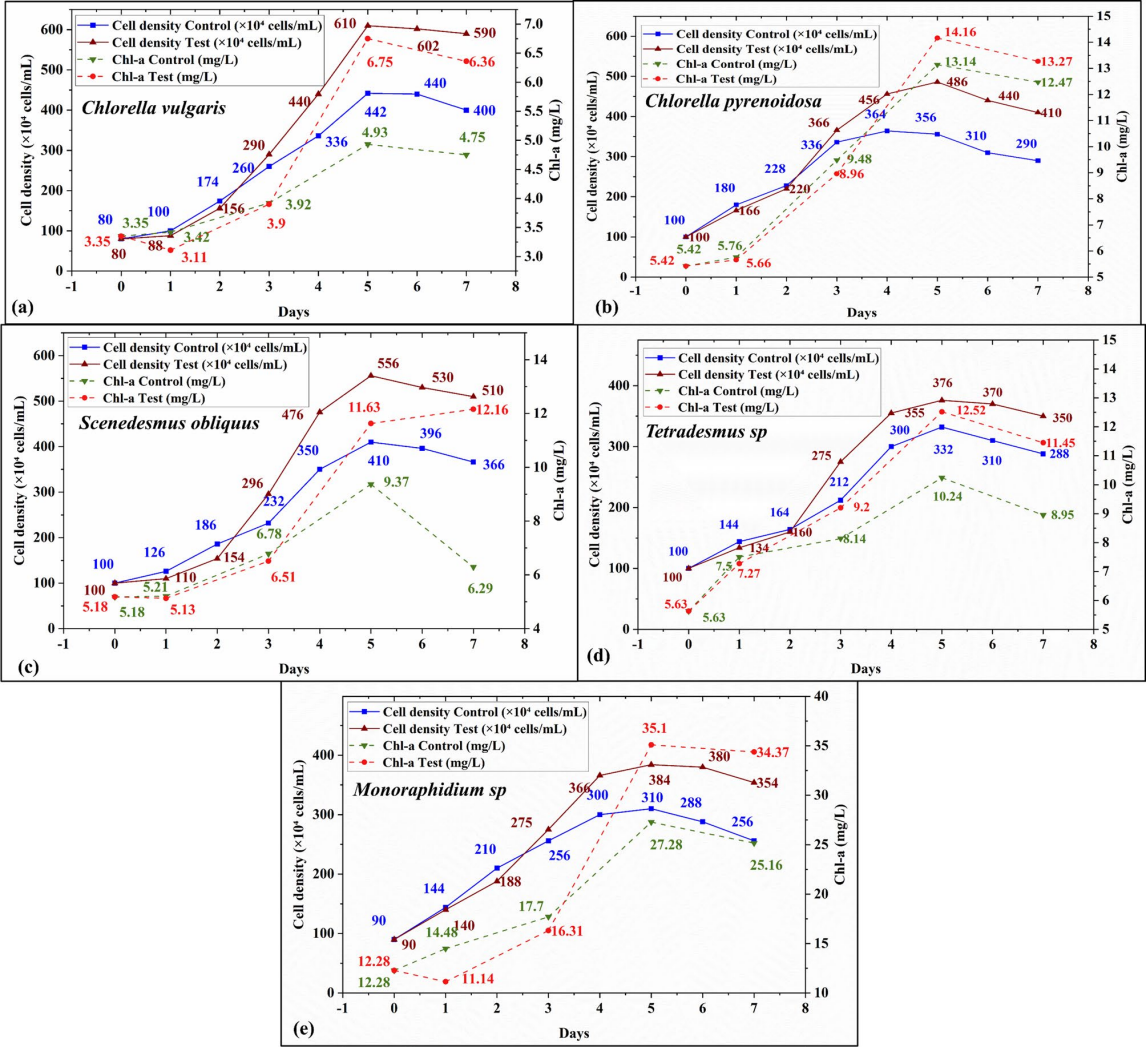
Effect of the binary mixture (CIP+NOR) on cell density and chl-a for (**a**) *Cv*, (**b**) *Cp,* (**c**) *So,* (**d**) *T,* and (**e**) *M.*

Algal cell density and chl-a were monitored every day until the algal species reached the death phase during their growth. Figure 6 compares the growth phase of algal species in the test runs with the control runs. The specific growth rate for control runs was 0.22 d^−1^ (*Cv*), 0.15 d^−1^ (*Cp*), 0.18 d^−1^ (*So*), 0.15 d^−1^ (*T*), and 0.14 d^−1^ (*M*) whereas for test runs it reached 0.28 d^−1^ (*Cv*), 0.2 d^−1^ (*Cp*), 0.23 d^−1^ (*So*), 0.17 d^−1^ (*T*), and 0.19 d^−1^ (*M*). The maximum growth rate was recorded for (*So*) 0.65 d^−1^ during day 4 of its exponential growth phase and the lowest growth rate was observed in the (*Cv*) (− 0.01 d^−1^) during day 6 (Fig. 6a), the negative growth rate indicates the decline in algal cell density due to the presence of the binary mixture, which affirms the acute toxicity assessment of (*Cv*) being the most sensitive species and (*So)* being the most tolerant species to the binary mixture of CIP+NOR. Dunnett tests were performed to compare the control runs and test runs in all the experimental runs to find the statistical significance. The generated *p*-values were 0.04 (*Cv*), 0.03 (*Cp*), 0.03 (*So*), 0.02 (*T*), and 0.04 (*M*). which were statistically significant at the (*p* < 0.05) level.

The growth pattern and the chl-a activity from the results indicate the algal ability to adapt to the stress conditions caused by the binary mixture of CIP+NOR. In all the species, the growth rate and chl-a were inhibited during the 2–3 days of its growth phase due to the acute stress. The cell density and chl-a activity increased after day 3 higher than the control runs affirming the growth-stimulating response of the algal species. For *Scenedesmus obliquus (So)* the photosynthetic activity was increasing even on day 7 (Fig. 6c), whereas in other species the chl-a activity dropped affirming the inhibitory test results of (*So*) adapting to the toxic conditions better than other selected species.

#### Removal mechanisms adapted by algal species

**Figure 7:**
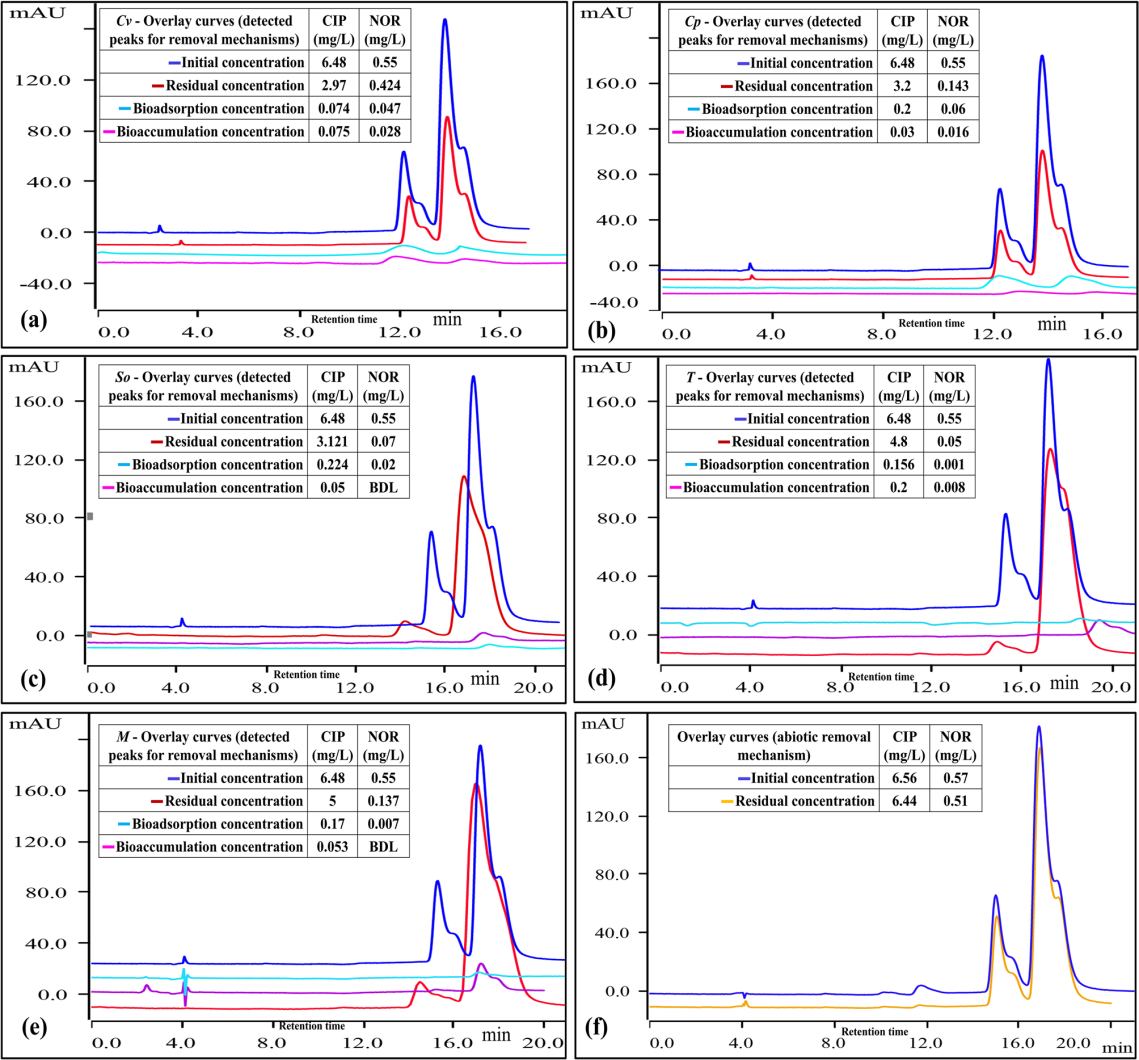
HPLC overlay detection peaks of binary composition (CIP+NOR) for (**a**) *Cv*, (**b**) *Cp,* (**c**) *So,* (**d**) *T,* (**e**) *M*, and (**f**) Abiotic removal.

Biotic removal (bioadsorption, bioaccumulation, and biodegradation) and Abiotic removal (photodegradation) are the two major contributions to the removal of CIP+NOR in the medium. Abiotic removal is considered to assess the actual biotic removal by algae in the medium. It has been observed that abiotic removal of NOR (10%) > CIP (1.9%) after 7 days (Fig. 7f) which indicates that NOR is more sensitive towards photodegradation in comparison with CIP^45^, however, the removal through abiotic conditions is negligible in comparison with the biotic removal.

Bioadsorption, bioaccumulation, and biodegradation are simultaneous events that occur in the algae cells for the assimilation of pollutants^53,54^. Bioadsorption is an extracellular mechanism that depends on the functional groups (proteins, cellulose, and hemicellulose) followed by bioaccumulation where the stress is counteracted by algae by the generation of reactive oxygen species (ROS) which leads to the biodegradation mechanism in algae^53,54^. Chl-a is a major light-harvesting pigment, during photosynthesis it releases protons inside the cell which enhances the production of reactive oxygen species (ROS) to counteract the stress caused by the pollutant^55^. This generated ROS in the algal cell takes part in the degradation of stress-causing pollutants in the algal medium^56^. The contribution of each mechanism in the removal of a binary mixture (CIP + NOR) is tabulated in Table 2. Figure 7 shows the consolidated overlay peak curves recorded in the HPLC analysis for the determination of removal mechanism adapted by different algal species

$$ chl{ - }a^{1} + hv \left( {light} \right) \to chl{ - }a^{3} \to chl{ - }a^{1} + singlet\, O_{2} + O_{2}^{ - } + OH + OOH $$

**Table 2:** CIP+NOR removal (%) and removal mechanism contribution (%) after 7 days.

Algal species	CIP+NOR	Total removal (%)	Removal mechanism contribution (%) for total removal			
			Photodegradation	Bioadsorption	Bioaccumulation	Biodegradation
*Cv*	CIP	55.13	3.54	2.05	2.12	92.26
	NOR	25.6	42.17	31.2	19.7	6.79
*Cp*	CIP	50.8	3.85	6.25	1.05	88.84
	NOR	74.9	14.46	14.93	3.38	67.2
*So*	CIP	52.4	3.73	6.44	1.52	88.3
	NOR	87.5	12.38	0.39	BDL	87.11
*T*	CIP	26.68	7.32	8.96	11.19	72.51
	NOR	88.69	15.99	0.19	1.46	82.35
*M*	CIP	23.8	8.2	1.05	3.3	87.4
	NOR	76.31	14.2	1.37	BDL	84.3

The removal efficiency of the binary mixture is in the order of *So* > *Cp* > *T* > *M* > *Cv* which is in line with the calculated 96 h EC_50_ order *So* > *Cp* > *T* > *M* > *Cv*. For *Chlorella vulgaris (Cv),* biodegradation (92%) is the major contributing removal mechanism for CIP, but NOR biodegradation is only 6% and 19% of NOR is accumulated inside the algal cell. The EC_50_ value indicated that Cv is more sensitive towards CIP compared to NOR, to counteract the stress caused, the reduction of CIP (55%) > NOR (25%) in the algal medium. Biodegradation contribution for the removal of CIP + NOR, in *Chlorella pyrenoidosa* (*Cp*) CIP (88%) > NOR (67%), *Scenedesmus obliquus* (*So*) CIP (88%) > NOR (87%), *Tetradesmus sp* (*T*) CIP (72%) < NOR (82%), and for *Monoraphidium sp* (*M*) CIP (87%) > NOR (84%). It is interesting to observe the biodegradation potential of *Monoraphidium sp* (*M*) is higher next to *Scenedesmus obliquus* (*So*) even though the acute toxicity studies showed that it is sensitive to the binary mixture.

Bioadsorption contribution for NOR is higher in the *Chlorella* species, 31% (*Cv*), and 14% (*Cp*) than in other species. This activity in *Chlorella* species can be evaluated by the log K_ow_ value of NOR (0.46) which is higher than CIP (0.26), studies have reported that the higher the value greater the adsorption of pollutants onto the cells^19,57^. The bioadsorption contribution (%) may also vary according to the species assimilation and acclimatizing potential of algal species. In *Tetradesmus sp* (*T*), biodegradation contribution for NOR is 82% > CIP (72%) and bioaccumulation for NOR is below the detection limit (BDL). In *Scenedesmus obliquus* (*So*), the bioadsorption contribution for CIP is 6% and NOR is 0.39%, and it can be noted that biodegradation contribution for NOR is 87% which is higher than the other species tested and bioaccumulation of NOR is below the deduction limit of the equipment. It is evident from the obtained results that *Scenedesmus obliquus* is effective in removal of CIP + NOR.

#### Biotransformed products inside *Scenedesmus obliquus*

**Figure 8:**
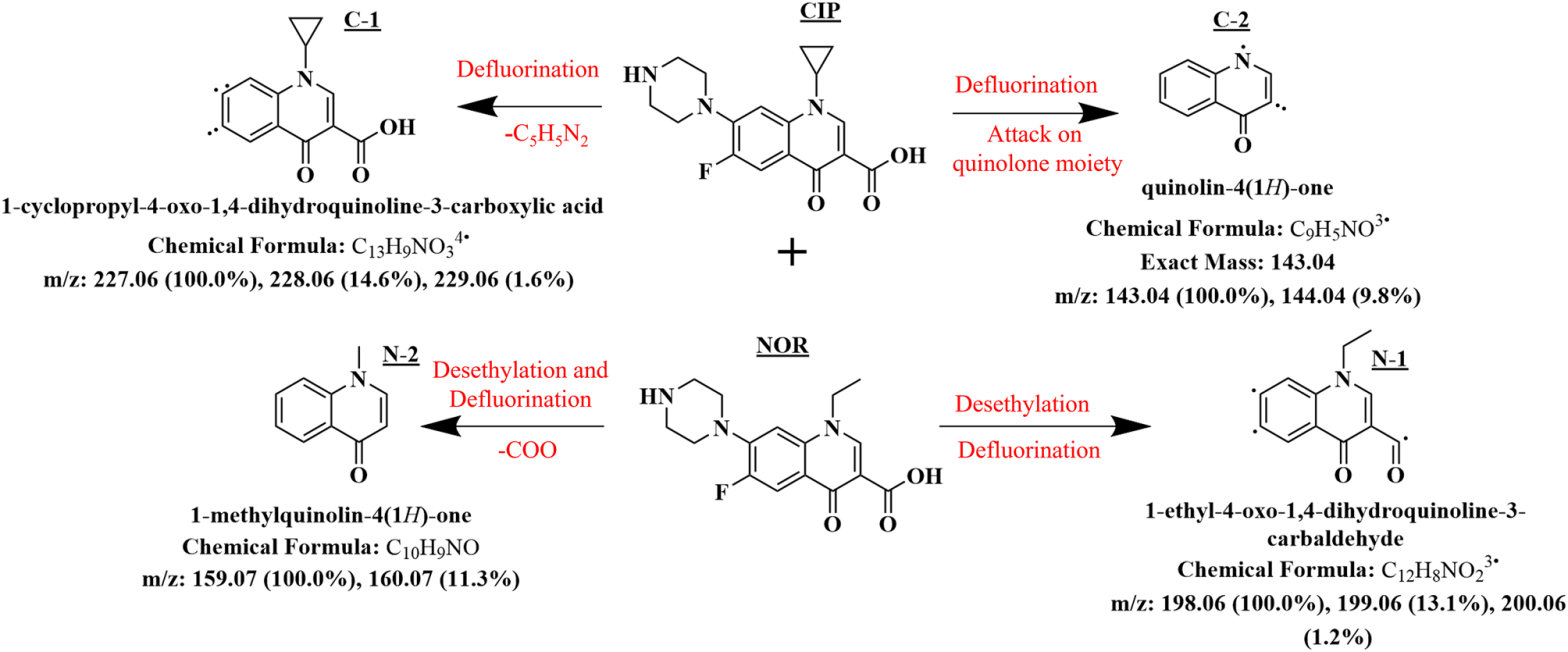
Possible biotransformation pathway of CIP+NOR in *Scenedesmus obliquus*.

From the experimental reports, it can be inferred that *Scenedesmus obliquus* performed better in terms of tolerance (96 h EC_50_) and removal efficiency. The biotransformed products inside the *Scenedesmus obliquus* are assessed by LC–MS analysis (Fig. 7) and the possible biotransformed pathway is depicted in Fig. 8. The ROS generated inside the algal cells attack the quinolone moiety (carboxyl group), F-atom, and piperazine to breakdown the parent compound into less toxic as a response to the stress induced by the binary mixture. Decomposition of piperazinyl by Cytochrome P450 enzyme and defluorination along with the fragmentation of C_5_H_5_N_2_ from the CIP by the ROS resulted in the formation of 1-cyclopropyl-4-oxo-1,4-dihydroquinoline-3-carboxylic acid (C1) and degradation of the quinoline moiety resulted in the formation of quinolin-4(1H)-one (C2). 1-ethyl-4-oxo-1,4-dihydroquinoline-3-carbaldehyde (N1) and 1-methylquinolin-4(1H)-one (N2) are the two biotransformed products formed as the result of desethylation and defluorination of NOR inside algal cells. The proposed degradation pathway in Fig. 8 is consistent with the literature survey^58–60^.

ECOSAR is employed to predict acute toxicity (96 h EC_50_) for biotransformed products, C1 (866 mg/L), C2 (156 mg/L), N1 (185 mg/L), and N2 (71 mg/L). The biotransformed products were less toxic compared to the parent compounds due to the loss of fluorine atom and piperazine ring from the compounds, as these substituents contribute to the antibacterial activity in Fluoroquinolone^61^. The presence of harmless biotransformed products inside *Scenedesmus obliquus* indicates the algal potential to treat these toxic compounds in wastewater matrices without the production of toxic by-products.

#### Biochemical composition assessment in all algal species

In all the experimental runs the accumulation of biomass (dry cell weight) was higher in the test runs than in the control runs. The biochemical composition (carbohydrates, lipids, and proteins) assessment was tabulated in Table 3. It can be noted from the table that *Scenedesmus obliquus* (*So*) has a higher lipid (35%), carbohydrate (18%), and protein (33%) accumulation compared to the control runs affirming the growth-stimulating response in the (*So*) species. In algal species (*Cv, Cp, T, and M*), being the sensitive species to the binary mixture the protein production decreased in comparison with the control runs. Algal cells convert the carbon source and CO_2_ into glyceraldehydes-3-phosphate (G3P) during photosynthesis by the action of chl-a activity^62^. G3P is responsible for the production of protein, carbohydrates, and lipid accumulation inside the cell, especially protein and lipids are the results of the generation of acetyl-CoA and pyruvate from G3P, when a toxic pollutant is accumulated inside algae cells, the cells counteract the stress conditions by increasing the production of lipids as a defense mechanism^63^. The increased production of lipids than the protein in all the algal species is due to the result of the stress induced by CIP+NOR.

**Table 3:** Effect of the binary mixture upon biochemical composition in algae after 7 days.

Algal species	Experimental runs	DCW (g/L)	Biochemical composition (%)		
			Protein	Carbohydrates	Lipids
*Cv*	Control	1.34	16.96	4.47	20.8
	Test	1.39	15.05	6.29	25.7
*Cp*	Control	2.2	25.4	6.4	17.3
	Test	2.4	23.3	8.13	21.2
*So*	Control	1.55	27.1	8.81	15.31
	Test	1.58	33.5	18.14	35.16
*T*	Control	1.24	25.7	7.3	19.1
	Test	1.27	25.02	11.55	28.61
*M*	Control	2.2	27.8	4.21	31.47
	Test	2.6	23	6.11	35.5

Comparing the biochemical composition of biomass in all the algal species. (*So*) has higher lipid accumulation with good removal efficiency and it can also tolerate the stress caused by the binary mixture*. Scenedesmus obliquus* is the most suitable candidate among these tested algal species in the treatment of antibiotic-polluted water with the advantage of valuable biomass production.

## Conclusion

In this experimental study, inhibitory tests were performed for *Chlorella vulgaris* (*Cv*), *Chlorella pyrenoidosa (Cp, Scenedesmus obliquus (So),* and *Tetradesmus sp (T),* and *Monoraphidium (M)* by spiking the algal medium with the binary mixture (CIP+NOR). Acute toxicity assessment revealed that *Scenedesmus obliquus* is the least sensitive algal species with 49% inhibition at 50 mg/L. Furthermore, removal studies revealed that *Scenedesmus obliquus* performed better in removing both pollutants with biodegradation as the major removal mechanism. LC–MS results revealed the biotransformed products are less toxic than the parent compound. Biochemical composition (protein, carbohydrates, and lipids) analysis revealed growth stimulating effect was observed higher in (*So*), this effect contributes an advantage in the utilization of biomass after the treatment for the production of value-added bioproducts.

*Scenedesmus obliquus* cannot be employed when the binary mixture concentration in the medium is higher than its tolerance level. However, there is a need to optimize and evaluate the performance of *Scenedesmus obliquus* in a pilot-scale study (real wastewater conditions). In addition, the formation of biotransformed products in the presence of other pollutants in the real wastewater need to be investigated.

## Data Availability

All the data generated or analyzed during this study are included in this article.
